# compareMS2 2.0: An Improved Software for Comparing Tandem Mass Spectrometry Datasets

**DOI:** 10.1021/acs.jproteome.2c00457

**Published:** 2022-09-29

**Authors:** Rob Marissen, Madhushri S. Varunjikar, Jeroen F. J. Laros, Josef D. Rasinger, Benjamin A. Neely, Magnus Palmblad

**Affiliations:** † Center for Proteomics and Metabolomics, Leiden University Medical Center, Postbus 9600, 2300 RC Leiden, The Netherlands; ‡ Institute of Marine Research, P.O. Box 1870 Nordnes, 5817 Bergen, Norway; § National Institute for Public Health and the Environment, 3720 BA Bilthoven, The Netherlands; ∥ Department of Human Genetics, Leiden University Medical Center, Postbus 9600, 2300 RC Leiden, The Netherlands; ⊥ National Institute of Standards and Technology, Charleston, South Carolina 29412, United States

**Keywords:** compareMS2, distance metric, molecular phylogenetics, tandem mass spectrometry, quality control

## Abstract

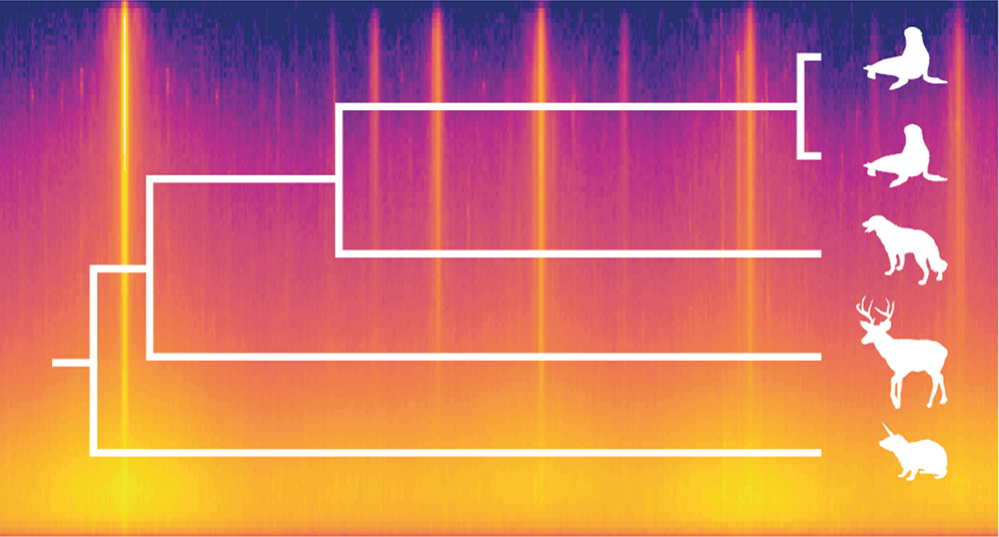

It has long been known that biological species can be identified from mass spectrometry data alone. Ten years ago, we described a method and software tool, compareMS2, for calculating a distance between sets of tandem mass spectra, as routinely collected in proteomics. This method has seen use in species identification and mixture characterization in food and feed products, as well as other applications. Here, we present the first major update of this software, including a new metric, a graphical user interface and additional functionality. The data have been deposited to ProteomeXchange with dataset identifier PXD034932.

## Introduction

A decade ago, Palmblad and Deelder^1^ first described a method for molecular phylogenetics based on direct comparison of tandem mass spectra. The method has since seen a range of applications, including food^2,3^ and feed^4−7^ species identification, quality control,^8^ and experimental design.^9^ Similar works include the DISMS2 library by Rieder and colleagues^10^ and MS1-only methods for “sequence-free” phylogenetics reviewed by Downard.^11^ Neely and Palmblad^12^ recently placed these methods in a larger historical context, going all the way back to the seminal comparison of separated tryptic peptides across species by Zuckerkandl, Jones, and Pauling in 1960.^13^ Here, we describe a new and significantly updated version of the original compareMS2 software, with several improvements, including a graphical user interface (GUI) controlling all steps of the analysis and dynamic phylogenetic tree display, a fully symmetric distance metric, and many additional filters and output options, which we describe in this technical note.

## Methods

### Symmetric Distance Measure

The original compareMS2 compared two sets of tandem mass spectra, e.g., those resulting from liquid chromatography–tandem mass spectrometry, by scanning one set and for each spectrum finding the best match in the other set (within precursor *m*/*z* and retention time tolerances). The results depended on which set was scanned, and the distance metric was only approximately symmetric. compareMS2 2.0 has a perfectly symmetric measure of the distance between sets of tandem mass spectra regardless of order of input. In this section, we describe this modified measure and some of its properties.

### Comparing Pairs of Spectra

The comparison between sets of tandem mass spectra starts with the comparison of pairs of spectra. There are many measures of spectral similarity. compareMS2 supports the cosine score (dot product) and spectral angle. By default, compareMS2 uses the cosine score, i.e., the cosine of the angle between the vector representations of the spectra, after normalizing both spectra to unit length:
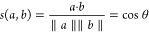
1where θ is the angle between the vector representations of the two spectra. Equation 1 is symmetric in *a* and *b*.

Optionally, compareMS2 can first scale spectra to reduce the influence of very intense peaks, e.g., by taking the square or cube root of all intensities. All peaks below a user-defined or automatically detected relative or absolute background can also be excluded from the similarity calculation.

### Comparing Sets of Spectra

compareMS2 2.0 defines the similarity between two sets of tandem mass spectra,  and  as follows. If for a spectrum *a*∈ we find a spectrum *b*∈ with *s*(*a*,*b*) greater than or equal to a minimum similarity threshold *s*_min_, we say that *a* has a similar spectrum in . We then define a subset ⊂, given , of all spectra in  with at least one similar spectrum in  as
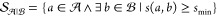
2and a corresponding subset ⊂ as
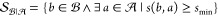
3

We then define a global similarity between sets  ≠ Ø and  ≠ Ø, *S*(,), as the average of the fraction of spectra in  with at least one similar spectrum in  and the fraction of spectra in  with at least one similar spectrum in :
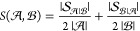
4where || denotes the cardinality, the number of elements, in a set . Though in some use cases it may be meaningful to define the similarity between two empty sets, i.e., LC-MS/MS datasets without tandem mass spectra, or the similarity between an empty and a non-empty set, we have chosen to leave these undefined and have the compareMS2 output reflect this. We believe this makes sense as a dataset without tandem mass spectra usually suggests something went wrong during measurement. Values can always be imputed after the compareMS2 runs, and rows with undefined values in the distance matrix can be excluded in subsequent analyses in most phylogenetic software.

From the symmetry of eq 4, we see that *S*(,) = *S*(,). We also note that both terms in eq 4 are non-negative, therefore *S*(,) ≥ 0. The maximum value of *S*(,) is 1 when all spectra in  have a similar spectrum in  and vice versa. The minimum value is 0 when  and  have no similar spectra. The smallest positive value of *S*(,) occurs when there is exactly one pair of similar spectra in  and :
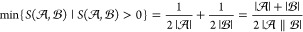
5

Finally, we arrive at the global distance measure, *D*(,), which we define as the inverse of *S*(,) minus one when *S*(,) is positive, and as the inverse of half of the smallest positive value of *S*(,) minus one when *S*(,) is zero:
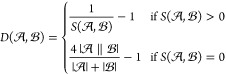
6Since *S*(,) is symmetric, *D*(,) is also symmetric. Note that *D*(,) → *∞* as || → *∞*∧|| → *∞*,and there are no similar spectra in  and . In the special case of  and  both containing a single spectrum, *D*(,) is 0 if the spectra are similar and 1 otherwise. The definition of the distance between sets with *S*(,) = 0 correspond to  and  having a hypothetical half matching spectrum. In most real-world use cases, both  and  would contain thousands of spectra.

Two co-directional spectra—spectra whose vector representations differ only by a factor—are considered identical by *s*. Therefore, datasets containing perfectly co-directional spectra would have a global similarity *S* = 1 and distance *D* = 0. Strictly speaking, *D* is not a metric in the mathematical sense, as the identity of indiscernibles (*D*(,) = 0 ⇔  = ) no longer holds after normalizing the spectra. This is by design, as the absolute intensities in a tandem mass spectrum depend not only on the peptide sequence and abundance, but also at which point or points during the chromatographic peak the peptide was selected for MS/MS, which is generally not reproducible.

As comparing all tandem mass spectra is computationally expensive, especially for large datasets. compareMS2 allows approximation of *D*(,) by only comparing a spectrum *a*∈ with those spectra *b*∈ that fall within user-defined windows of retention time or scan number, and precursor *m*/*z*.

### compareMS2 Pipeline

**Figure 1:**
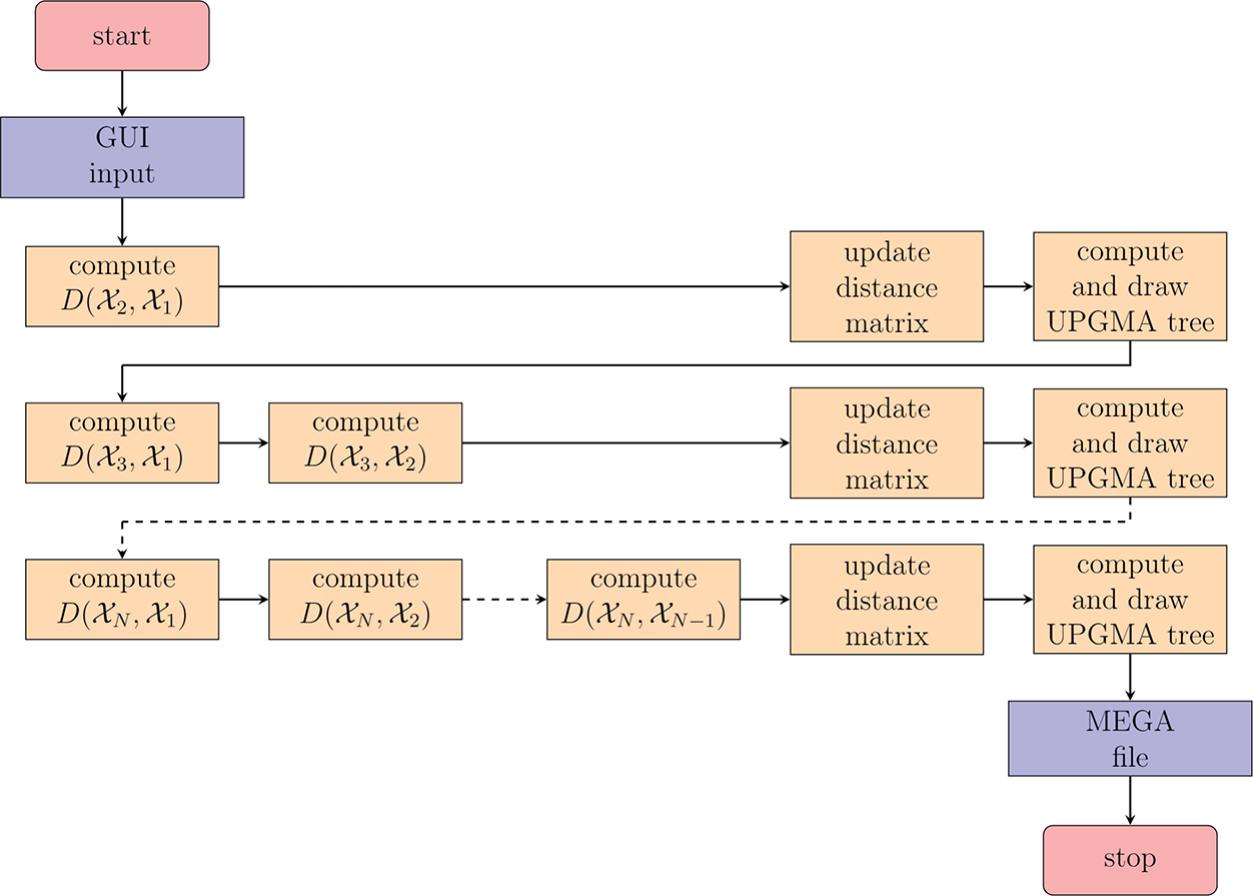
CompareMS2 2.0 workflow, orchestrated by the graphical user interface. After parsing and checking the input parameters, ensuring all files are present and in the correct format, compareMS2 performs (*N*^2^ – *N*)/2 pairwise comparisons of *N* datasets using the symmetric distance measure described below, or *N*^2^ – *N* comparisons if the original measure is used. After each row is completed, compareMS2 updates the (strictly triangular) distance matrix and generates a new tree. This allows the user to monitor progress and terminate and restart the run if necessary. If the original measure is used, compareMS2 by default creates both the strictly upper and lower triangular distance matrices (these can be averaged in phylogenetics software such as MEGA).

compareMS2 takes as minimum input a directory of MGF files to be compared. We choose MGF as the default input format, as it is convenient for storing MS2-only data and the MGF files can easily be filtered, split or combined, which may be useful in some applications of compareMS2, such as when fractionating samples or removing nonpeptide spectra. Most vendor software as well as msconvert^14^ can convert raw data or mzML files to MGF. To provide faster feedback to the user, compareMS2 2.0 interleaves distance matrix calculations, updates and displays a phylogenetic tree as each row of the distance matrix is completed (Figure 1). With the default symmetric metric, this matrix is triangular, hence the tree is updated rapidly in the beginning, after the first comparison, and then again after the next two comparisons etc. Version 2.0 also provides additional functionality, such as recording a quality control metric for each dataset (by default the number of tandem mass spectra in the dataset) and a filter to compare only the top-*N* most intense tandem mass spectra from each dataset. The datasets can be compared in alphabetical, size or random order. By default, compareMS2 outputs a MEGA (.meg) file, but Newick and NEXUS formats are also supported.

### compareMS2 GUI

**Figure 2:**
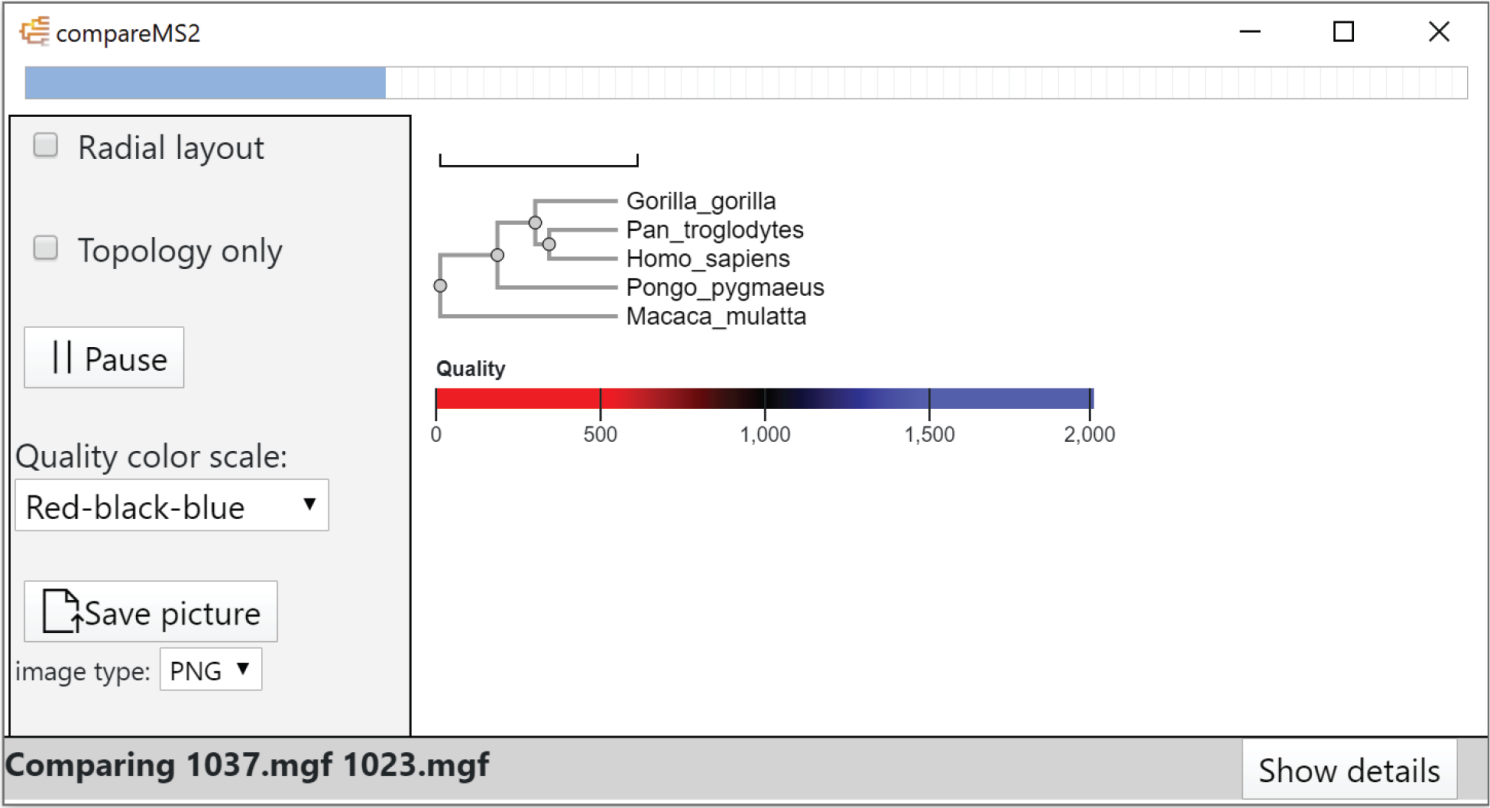
compareMS2 2.0 GUI, showing the output panel from the beginning of an analysis of 24 datasets, each containing 1000 tandem mass spectra, from six primate species for a total of (24^2^ – 24)/2 = 276 comparisons. With default parameters, these comparisons take 3 min on a PC with an Intel Xeon W-2135 CPU running at 3.70 GHz. The node text color in the tree represents data quality, the default metric being the number of tandem mass spectra per species.

Technically, compareMS2 2.0 combines two software tools, which can also be run individually on the command line. The first component compares two datasets, e.g., from LC-MS/MS. The second component takes several such comparisons, combines samples from the same biological species, and computes a distance matrix. The graphical user interface (Figure 2) was designed to be simple to use, hiding most of the internal complexity of compareMS2, including the interleaved execution order of the two components (Figure 1).

### Source Code and Availability

The compareMS2 source code can freely be downloaded from https://github.com/524D/compareMS2. On Windows, the software can be installed using a simple installer. compareMS has been tested on Windows 10, Ubuntu 20.04 Linux and MacOS 12. The GUI is based on Electron (https://www.electronjs.org/) and is written in Javascript, HTML, and CSS. It uses the phylotree.js library^15^ to render the graphical tree representation. Conversion of the distance matrix into Newick format uses the UPGMA method is and is also implemented in JavaScript. The distance computation and distance matrix creation are performed by two command-line programs written in C. These can be used to run compareMS without the GUI. Source code and prebuild executables of the command-line tools can be found in the external_binaries directory of the compareMS2 repository.

### Experimental Features

As compareMS2 provides a basic framework for comparing tandem mass spectra across datasets, we have begun to add experimental features to help visualize such comparisons. The first of these experimental outputs is a two-dimensional histogram of precursor *m*/*z* difference and spectral similarity for all comparisons of spectra between two datasets. These features will only be available on the command-line, and require additional software such as R to generate figures, but allow for example correlating spectral similarity with precursor mass difference. Scripting examples in R are available on https://osf.io/jey28/.

### Testing

To demonstrate the features and performance of compareMS2 2.0, we used previously published amaZon ion trap (Bruker Daltonics) and Orbitrap Fusion Lumos (Thermo Fisher Scientific) data from primate sera and an *E. coli* lysate.^1,12^ In addition, we used new data acquired on the same Orbitrap instrument and as described in^12^ from California sea lion (*Zalophus californianus*), dog (*Canis lupus familiaris*), rock hyrax (*Procavia capensis*), and white-tailed deer (*Odocoileus virginianus*) sera. The mass spectrometry proteomics data have been deposited to the ProteomeXchange Consortium via the PRIDE^16^ partner repository with the dataset identifier PXD034932 and 10.6019/PXD034932. Phylogenetic trees were generated by compareMS2 and MEGA11^17^ using default parameters for both (for compareMS2 maximum precursor mass difference 2.05, score cutoff 0.8, minimum basepeak intensity 10000, minimum total ion current 0, maximum retention time difference 60, start retention time 0, end retention time 100000, maximum scan number difference 10000, start scan 1, end scan 1000000, scaling 0.5, noise 10, version of set distance metric 2, version of QC metric 0, compare only the N most intense spectra set to “All”, output format “MEGA”,and compare order “Smallest-largest first”, and for MEGA11 “Lower Left Matrix” and “Pairwise Distance” input data for UPGMA Phylogeny Analysis).

## Results and Discussion

The compareMS2 2.0 GUI (Figure 2) displays a phylogenetic tree with a quality metric mapped to a continuous or divergent color gradient, the tree being continuously updated to provide real-time feedback to the user. This allows executions to be paused or terminated at any stage, which may be useful for large jobs. For example, comparing 100 LC-MS/MS datasets require 4950 pairwise comparisons, taking several hours. But already after six pairwise comparisons of four datasets, trees can be quite informative and reveal if there is an issue with the input files or parameters.

**Figure 3:**
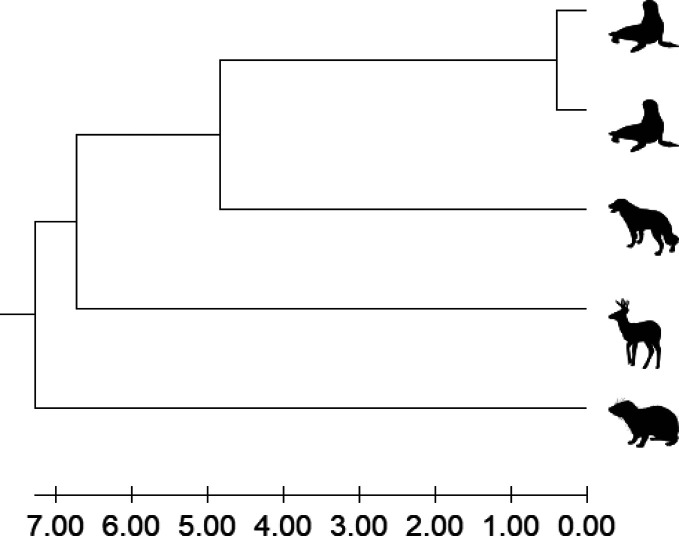
Phylogenetic analysis in MEGA11 based on Orbitrap Fusion Lumos LC-MS/MS datasets of sera from (top to bottom) two California sea lions (*Zalophus californianus*), dog (*Canis lupus familiaris*), white-tailed deer (*Odocoileus virginianus*), and rock hyrax (*Procavia capensis*). The evolutionary history was inferred using the UPGMA method.^18^ The optimal tree is shown and drawn to scale, with branch lengths in the same units as those generated by compareMS2 and used to infer the phylogenetic tree. Taxon images are from PhyloPic.

Using the five new serum datasets, each containing between 42,629 and 47,626 tandem mass spectra, we could reconstruct the correct phylogenetic tree in compareMS2 and MEGA11 (Figure 3). The 10 pairwise comparisons in compareMS2 took 40 min with default parameters on a PC with an Intel Xeon W-2135 CPU running at 3.70 GHz. The analyses can be accelerated by comparing spectra within a more narrow *m*/*z* window than the default value of 2.05. Each comparison is independent, so in principle the problem is embarrassingly parallel.

**Figure 4:**
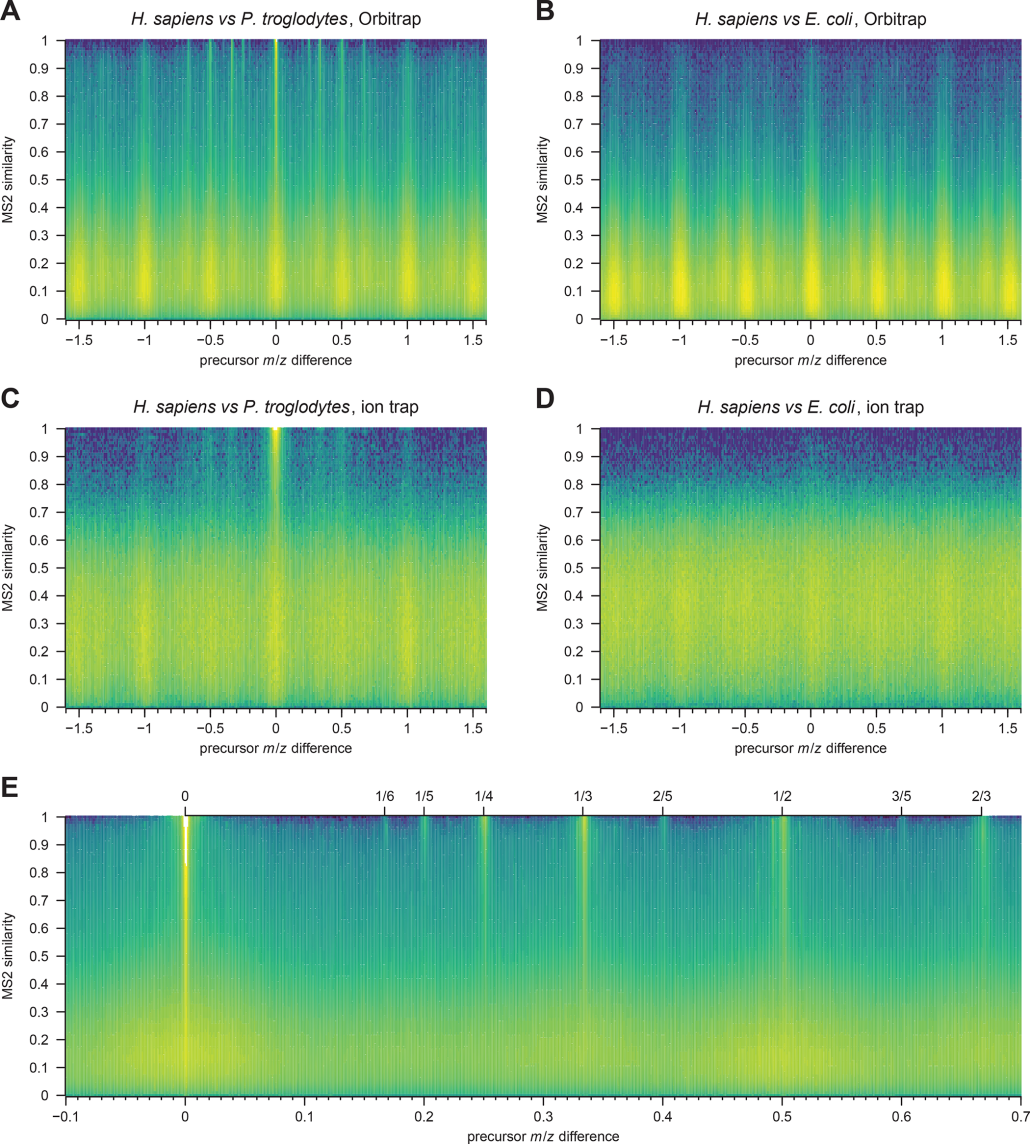
Similarity of tandem mass spectra as a function of precursor *m*/*z* difference in Orbitrap Fusion Lumos (A,B) and amaZon ion trap data (C,D), comparing similar (human and chimpanzee sera) and dissimilar (human serum and *E. coli*) samples. Panels A and B compare two LC-MS/MS runs, and panels C and D compare four runs per species (16 comparisons). Similar spectra have precursor *m*/*z* differences near zero or a near a rational number corresponding to the isotope error at a specific charge state (shown more clearly in panel E, generated from 8 Orbitrap human serum datasets).

To test one of the experimental features, we compared the similarity between tandem mass spectra as a function of precursor *m*/*z* difference for comparisons between two closely related species - human and chimpanzee - as well as two species with few shared tryptic peptides—human and *E. coli* (Figure 4). These comparisons reveal information on spectral similarity, but also on mass measurement precision, charge states and isotope errors *before* and independent of any database search, where such parameters typically have to be provided. In these datasets, charge states up to [M + 6H]^6+^ and isotope errors up to at least 3 Da are observed. The analysis can also be used to estimate suitable parameters for compareMS2, e.g., *m*/*z* windows and spectral similarity thresholds. We also observe some unexpected side bands most noticeable at 1/2 and 1/3 Da, but not near zero, in the Orbitrap data. These bands are also seen in comparisons of spectra within individual datasets.

When combined with posterior error probability estimators such as PeptideProphet^19^ or Percolator,^20^ spectral similarity measures can in principle be converted into probabilities for any pair of spectra being derived from the same or closely related analytes. When searching spectral libraries, the probability that a query spectrum matches the library spectrum is multiplied with the original probability that library spectrum was correctly identified to estimate the probability the query spectrum is correctly matched to a peptide or other analyte. The compareMS2 software uses the spectral similarity in eq 1 to calculate the overlap between sets of tandem mass spectra without regard to their identification to a specific analyte.

Naïvely, one may attempt to use something like the Jaccard similarity, *J*, defined as the cardinality of the intersection divided by the cardinality of the union
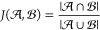
7However, no two spectra are exactly the same. If the criterion for considering two spectra identical (as in derived from the same peptide) for the purpose of calculating |∩| and |∪| is too strict, then one will underestimate |∩| and overestimate |∪|. If the criterion is too lax, then one overestimates |∩| and underestimates |∪|. In either case, the errors would multiply, making the Jaccard similarity very sensitive to the precise definition of when two spectra are considered identical. Even more problematic is the intransitive nature of this identity, which is exacerbated by chimeric spectra—spectra that are superpositions of two or more peptide tandem mass spectra. Briefly, a pure spectrum from peptide *P* can be considered identical to a chimeric spectrum with a small contribution from a second, cofragmenting peptide *Q*, which in turn is identical to a chimeric spectrum with slightly larger contribution from peptide *Q*, and so on, eventually ending up with the pure spectrum of peptide *Q*, which can be very different from the original spectrum from peptide *P*, just like messages in a game of telephone. This is why exercises clustering large numbers of tandem mass spectra based on spectral similarity tend to produce large globs of spectra rather than a distinct cluster for each peptide.

## Conclusions

compareMS2 compares sets of tandem mass spectra to each other rather than to predicted spectra of specific peptides as when identifying proteins from tandem mass spectra. We have used examples from molecular phylogenetics, but many other uses have been demonstrated, including food and feed identification, mixture analysis and experimental design. compareMS2 may also be used data quality control - comparing large numbers of datasets prior to database search and protein quantitation to detect outliers and possible batch effects. The visualization of spectral similarity as a function of precursor mass difference gives another window into the data, and can suggest parameters for database searches a priori. We make compareMS2 freely available as open source and provide an automatic installer for Microsoft Windows in hope that it may be as useful to others as it has been for us.

## References

[ref1] Palmblad M.; Deelder A. M. Molecular phylogenetics by direct comparison of tandem mass spectra. Rapid Commun. Mass Spectrom. 2012, 26, 728–732. 10.1002/rcm.6162. 22368051

[ref2] Wulff T.; Nielsen M. E.; Deelder A. M.; Jessen F.; Palmblad M. Authentication of fish products by large-scale comparison of tandem mass spectra. J. Proteome Res. 2013, 12, 5253–5259. 10.1021/pr4006525. 24032411

[ref3] Ohana D.; Dalebout H.; Marissen R.; Wulff T.; Bergquist J.; Deelder A.; Palmblad M. Identification of meat products by shotgun spectral matching. Food chemistry 2016, 203, 28–34. 10.1016/j.foodchem.2016.01.138. 26948585

[ref4] Rasinger J.; Marbaix H.; Dieu M.; Fumière O.; Mauro S.; Palmblad M.; Raes M.; Berntssen M. Species and tissues specific differentiation of processed animal proteins in aquafeeds using proteomics tools. Journal of proteomics 2016, 147, 125–131. 10.1016/j.jprot.2016.05.036. 27268957

[ref5] Belghit I.; Lock E.-J.; Fumière O.; Lecrenier M.-C.; Renard P.; Dieu M.; Berntssen M. H. G.; Palmblad M.; Rasinger J. D. Species-Specific Discrimination of Insect Meals for Aquafeeds by Direct Comparison of Tandem Mass Spectra. Animals: an open access journal from MDPI 2019, 9, 222 10.3390/ani9050222. 31067722 PMC6562778

[ref6] Belghit I.; et al. Future feed control – Tracing banned bovine material in insect meal. Food Control 2021, 128, 108183 10.1016/j.foodcont.2021.108183.

[ref7] Varunjikar M. S.; Belghit I.; Gjerde J.; Palmblad M.; Oveland E.; Rasinger J. D. Shotgun proteomics approaches for authentication, biological analyses, and allergen detection in feed and food-grade insect species. Food Control 2022, 137, 108888 10.1016/j.foodcont.2022.108888.

[ref8] van der Plas-Duivesteijn S. J.; Mohammed Y.; Dalebout H.; Meijer A.; Botermans A.; Hoogendijk J. L.; Henneman A. A.; Deelder A. M.; Spaink H. P.; Palmblad M. Identifying proteins in zebrafish embryos using spectral libraries generated from dissected adult organs and tissues. J. Proteome Res. 2014, 13, 1537–1544. 10.1021/pr4010585. 24460240

[ref9] van der Plas-Duivesteijn S. J.; Wulff T.; Klychnikov O.; Ohana D.; Dalebout H.; van Veelen P. A.; de Keijzer J.; Nessen M. A.; van der Burgt Y. E. M.; Deelder A. M.; Palmblad M. Differentiating samples and experimental protocols by direct comparison of tandem mass spectra. Rapid communications in mass spectrometry: RCM 2016, 30, 731–738. 10.1002/rcm.7494. 26864526

[ref10] Rieder V.; Blank-Landeshammer B.; Stuhr M.; Schell T.; Biß K.; Kollipara L.; Meyer A.; Pfenninger M.; Westphal H.; Sickmann A.; Rahnenführer J. DISMS2: A flexible algorithm for direct proteome- wide distance calculation of LC-MS/MS runs. BMC bioinformatics 2017, 18, 148 10.1186/s12859-017-1514-2. 28253837 PMC5335755

[ref11] Downard K. M. Sequence-Free Phylogenetics with Mass Spectrometry. Mass Spectrom. Rev. 2020, 41, 3–14. 10.1002/mas.21658. 33169385

[ref12] Neely B. A.; Palmblad M. Rewinding the Molecular Clock: Looking at Pioneering Molecular Phylogenetics Experiments in the Light of Proteomics. J. Proteome Res. 2021, 20, 4640–4645. 10.1021/acs.jproteome.1c00528. 34523928 PMC8491155

[ref13] Zuckerkandl E.; Jones R.; Pauling L. A Comparison of Animal Hemoglobins by Tryptic Peptide Pattern Analysis. Proc. Natl. Acad. Sci. U.S.A. 1960, 46, 1349–1360. 10.1073/pnas.46.10.1349. 16590757 PMC223050

[ref14] Chambers M. C.; et al. A cross-platform toolkit for mass spectrometry and proteomics. Nature biotechnology 2012, 30, 918–920. 10.1038/nbt.2377. PMC3471674 23051804

[ref15] Shank S. D.; Weaver S.; Kosakovsky Pond S. L. phylotree.js - a JavaScript library for application development and interactive data visualization in phylogenetics. BMC bioinformatics 2018, 19, 276 10.1186/s12859-018-2283-2. 30045713 PMC6060545

[ref16] Perez-Riverol Y.; Bai J.; Bandla C.; García-Seisdedos D.; Hewapathirana S.; Kamatchinathan S.; Kundu D.; Prakash A.; Frericks-Zipper A.; Eisenacher M.; Walzer M.; Wang S.; Brazma A.; Vizcaíno J. The PRIDE database resources in 2022: a hub for mass spectrometry-based proteomics evidences. Nucleic Acids Res. 2022, 50, D543–D552. 10.1093/nar/gkab1038. 34723319 PMC8728295

[ref17] Tamura K.; Stecher G.; Kumar S. MEGA11: Molecular Evolutionary Genetics Analysis Version 11. Mol. Biol. Evol. 2021, 38, 3022–3027. 10.1093/molbev/msab120. 33892491 PMC8233496

[ref18] Sneath P.; Sokal R. Numerical Taxonomy: The Principles and Practice of Numerical Classification; Freeman: San Francisco, 1973.

[ref19] Keller A.; Nesvizhskii A. I.; Kolker E.; Aebersold R. Empirical Statistical Model To Estimate the Accuracy of Peptide Identifications Made by MS/MS and Database Search. Anal. Chem. 2002, 74, 5383–5392. 10.1021/ac025747h. 12403597

[ref20] Käll L.; Canterbury J. D.; Weston J.; Noble W. S.; MacCoss M. J. Semi-supervised learning for peptide identification from shotgun proteomics datasets. Nat. Methods 2007, 4, 923–925. 10.1038/nmeth1113. 17952086

